
JOURNAL INFORMATION
==============================
NLM Title Abbreviation: Daniels Tex Med J
NLM Journal ID: 0006376

Daniel's Texas Medical Journal

ISSN: 0892-8487
Publisher: Biomedical Journal Digitization Project

ARTICLE INFORMATION
==============================
PMCID: PMC9176589
Article version: 1
Subjects: Book Notices

The International Medical Annual

And Practitioner’s Index for 1893

Print publication date: 1893 May
Volume: 8
Issue: 11
First page: 485
Last page: 485
License: This work is free of known copyright restrictions. For more information, please see PMC Back Issue Digitization.
License URL: https://creativecommons.org/publicdomain/mark/1.0/

==============================

### Book Notices.

THE INTERNATINAL MEDICAL ANNUAL and Practitioner's Index for 1893. Edited by a corps of thirty-eight department editors—European and American—specialists in their several departments. P. W. Williams, M. D., Secretary of Staff. 626 octavo pages. Illustrated. $2.75. E. B. Treat, Publisher, 5 Cooper Union, New York.

The eleventh yearly issue of this one-volume reference book is to hand; and it richly deserves and perpetuates the enviable reputation which its predecessors have made, for selection of material, accuracy of statement and great usefulness. The corps of department editors is representative in every respect. Numerous illustrations—many of which are in colors—make the "Annual" more than ever welcome to the Profession, as providing, at a reasonable outlay, the handiest and best resumé of Medical Progress yet offered.

The arrangement of the work is alphabetical, and with its complete index makes it a reference book of rare worth.

In short, the "Annual" is what it claims to be—a recapitulation of the year's progress in medicine, serving to keep the practitioner abreast of the times with reference to the medical literature of the world. Price, the same as in previous years—$2.75.

### Publishers' Notes.

To Cure Leucorrhea is easy enough, if you know how. We will tell you how to do it. Use Chloro-Phénique. See?

The Celebrated Baltimore College of Physicians and Surgeons has an advertisement with us again. Please read it, and write to Dr. Opie for further information.

The Bedford Mineral Springs, Pennsylvania, are advertised in this issue. We suggest to our readers to write to the address given, and obtain full information and testimonials.

Following Mr. Howells' lead, apparently, both Frank R. Stockton and Mrs. Frances Hodgson Burnett have gone over to *The Ladies' Home Journal*, and the most important works by these authors upon which they are now engaged, will shortly see publication in this magazine.

We have had great satisfaction purchasing goods, supporters, etc., of G. W. Flavell & Bro., 1005 Spring Garden street, Philadelphia, Pa. They commenced in a very moderate way and by strict fidelity and honest goods have worked their way up to a large business.—*Medical World*, April, 1893.
